# Supplementary material for: Gut microbiota-derived tryptamine and phenethylamine impair insulin sensitivity in metabolic syndrome and irritable bowel syndrome
Source: Nat Commun. 2023 Aug 17;14:4986. doi: 10.1038/s41467-023-40552-y (PMC10435514; doi:10.1038/s41467-023-40552-y)
Supplement: Supplementary file 1 — Supplementary Information [file 41467_2023_40552_MOESM1_ESM.pdf]

Supplemental Information

Gut microbiota-derived tryptamine and phenethylamine impair insulin sensitivity in metabolic syndrome and irritable bowel syndrome

Lixiang Zhai[1,2,†], Haitao Xiao[3,†], Chengyuan Lin[1,†], Hoi Leong Xavier Wong[2,†], Yan Y. Lam[1,†], Mengxue Gong[4], Guojun Wu[5], Ziwan Ning[1,2], Chunhua Huang[1,2], Yijing Zhang[2], Chao Yang[6], Jingyuan Luo[1,2], Lu Zhang[6], Ling Zhao[7], Chenhong Zhang[4], Johnson Yiu-Nam Lau[2], Aiping Lu[2], Lok-Ting Lau[2], Wei Jia[8,9,*], Liping Zhao[5,*], Zhao-Xiang Bian[1,2,*]

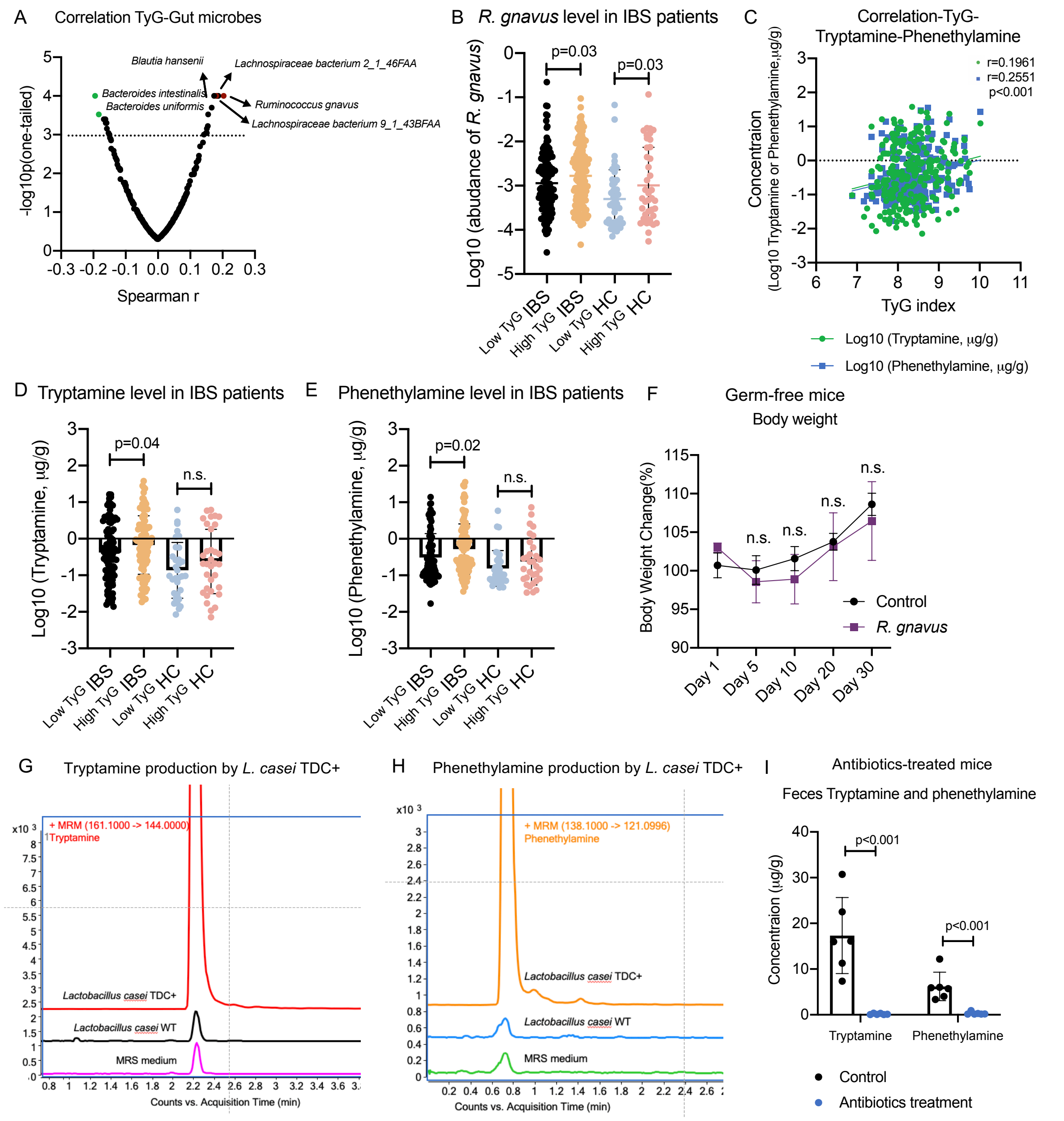

Figure.S1 Positive association and causality between *R. gnavus*-derived tryptamine/phenethylamine and insulin resistance in irritable bowel syndrome, Related to Figure.1

(**A**) Spearman r and p-values (-log10p) plot against gut bacteria species abundances and TyG level in human participants (n=412) (one-tailed test). (**B**) *R. gnavus* abundances in IBS-D patients (n=290) and healthy control (HC, n=89) subjects with different TyG indexes using a 50% cut-off value (two-tailed test). (**C**) Spearman's correlation between relative abundances

of *R. gnavus* with TyG level in human participants (n=412) (one-tailed test). (**D-E**) Tryptamine and phenethylamine levels in IBS-D patients (n=290) and HC subjects (n=89) with different TyG index using a 50% cut-off value (two-tailed test). (**F**) Body weight changes in germ-free mice following colonization of *R. gnavus* ATCC 29149 (n=6 per group) (two-tailed test). **(G-H)** LC-MS chromatogram of tryptamine and phenethylamine level in MRS culture medium of *L. casei* TDC+ and *L. casei* vector control (WT). Differences in body weight changes were determined by two-way ANOVA. Data are presented as mean$\pm$S.D. **(I)** Tryptamine and phenethylamine levels in fecal samples of normal mice from the control group (without antibiotics treatment, n=6) and the group treated with antibiotics mixture (n=12) (two-tailed test). Differences of phenethylamine and tryptamine levels in serum and fecal samples were analyzed by one-tailed student t-tests. Data are presented as mean $\pm$ S.D.

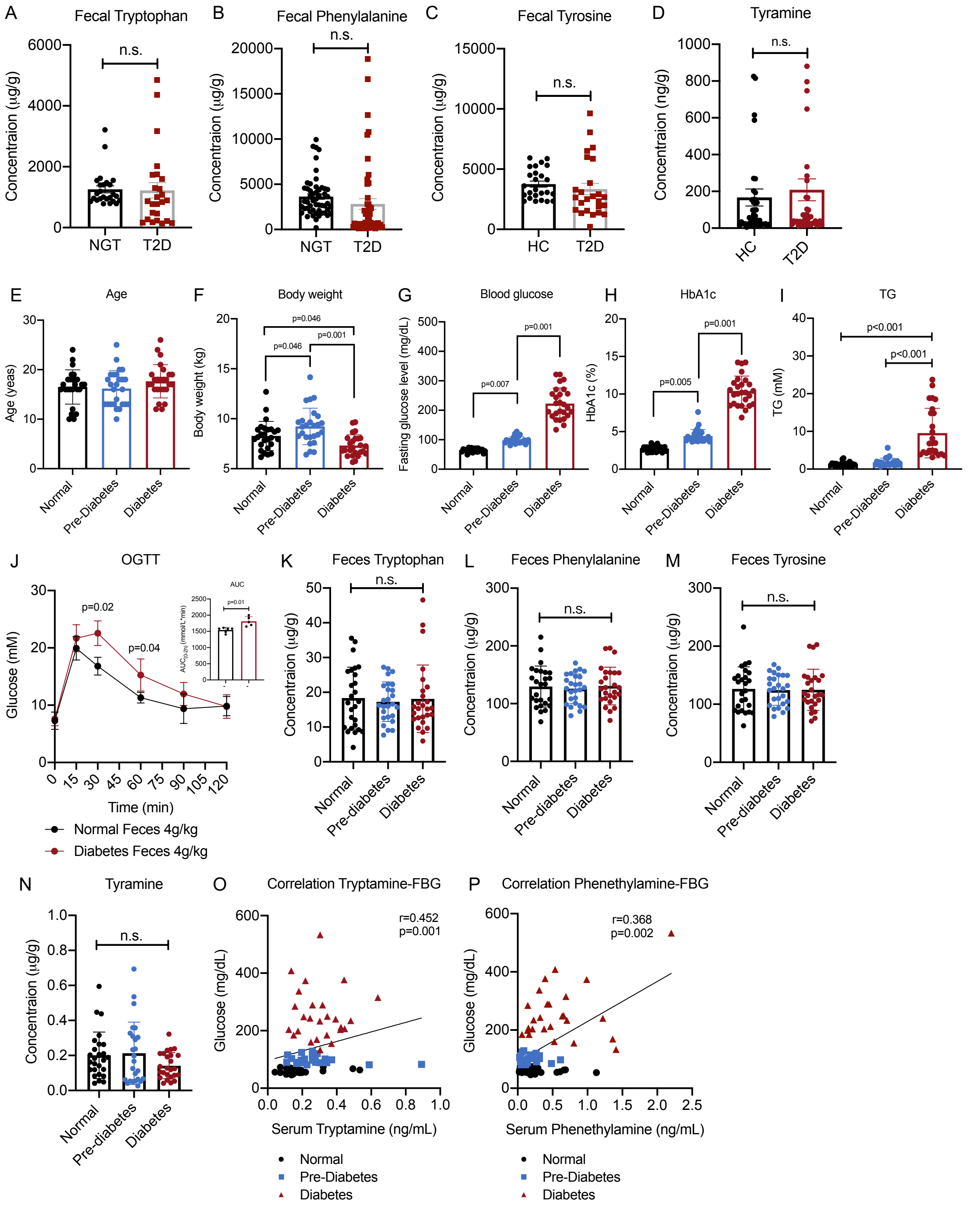

Figure.S2 Tryptamine and phenethylamine are positively correlated with glucose intolerance in patients with type 2 diabetes and monkeys with spontaneous diabetes, Related to Figure.2

(A-D) Tryptophan and phenylalanine (substrates of tryptamine and phenethylamine) levels and tyrosine and tyramine in fecal samples from individuals with or without T2D (n=25 subjects with NGT, n=25 patients with T2D). **(E-I)** Age, body weight, FBG, HbA1c and TG levels in monkeys without or with pre-diabetic or diabetes (n=26/per group). **(J)** OGTT index in HFD-fed mice after treatment with fecal suspension from normal monkeys and diabetes monkeys once per day for 5 days (n=6/group). (**K-M**) Tryptophan and phenylalanine (substrates of tryptamine and phenethylamine) levels and tyrosine and tyramine in fecal samples of monkeys without or with pre-diabetic or diabetes (n=26/per group). **(O-P)** Spearman's correlation between serum tryptamine and phenethylamine levels with FBG level in monkeys with or without pre-diabetes and diabetes (n=26/group). Differences of phenethylamine and tryptamine levels in fecal and serum samples were analyzed by one-tailed student t-tests. Differences of age, body weight, FBG, HbA1c, TG and OGTT indexes in monkeys were analyzed by two-tailed ordinary one-way ANOVA. Data are presented as mean $\pm$ S.D.

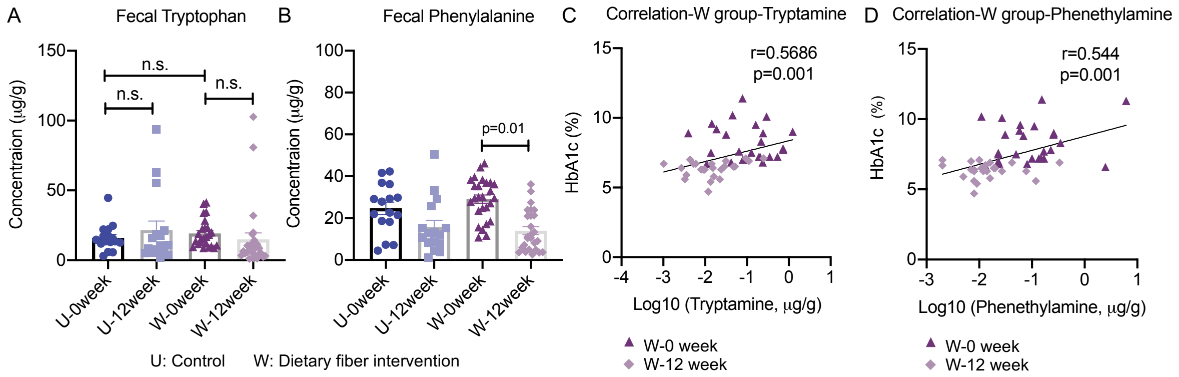

Figure.S3 Tryptamine and phenethylamine are negatively correlated with the improvement of insulin sensitivity in dietary fiber-treated patients with type 2 diabetes, Related to Figure.3

(A-B) Tryptophan and phenylalanine levels in fecal samples of individuals with or without type 2 diabetes (n=25 subjects with NGT, n=25 patients with T2D). **(C-D)** Spearman's

correlation analysis between fecal tryptamine and phenethylamine level and HbA1c index in T2D subjects consuming a high fiber diet (W group; n=27). Data are presented as mean±S.D. *P* values were determined by one-tailed test of ordinary one-way ANOVA or student's t-test.

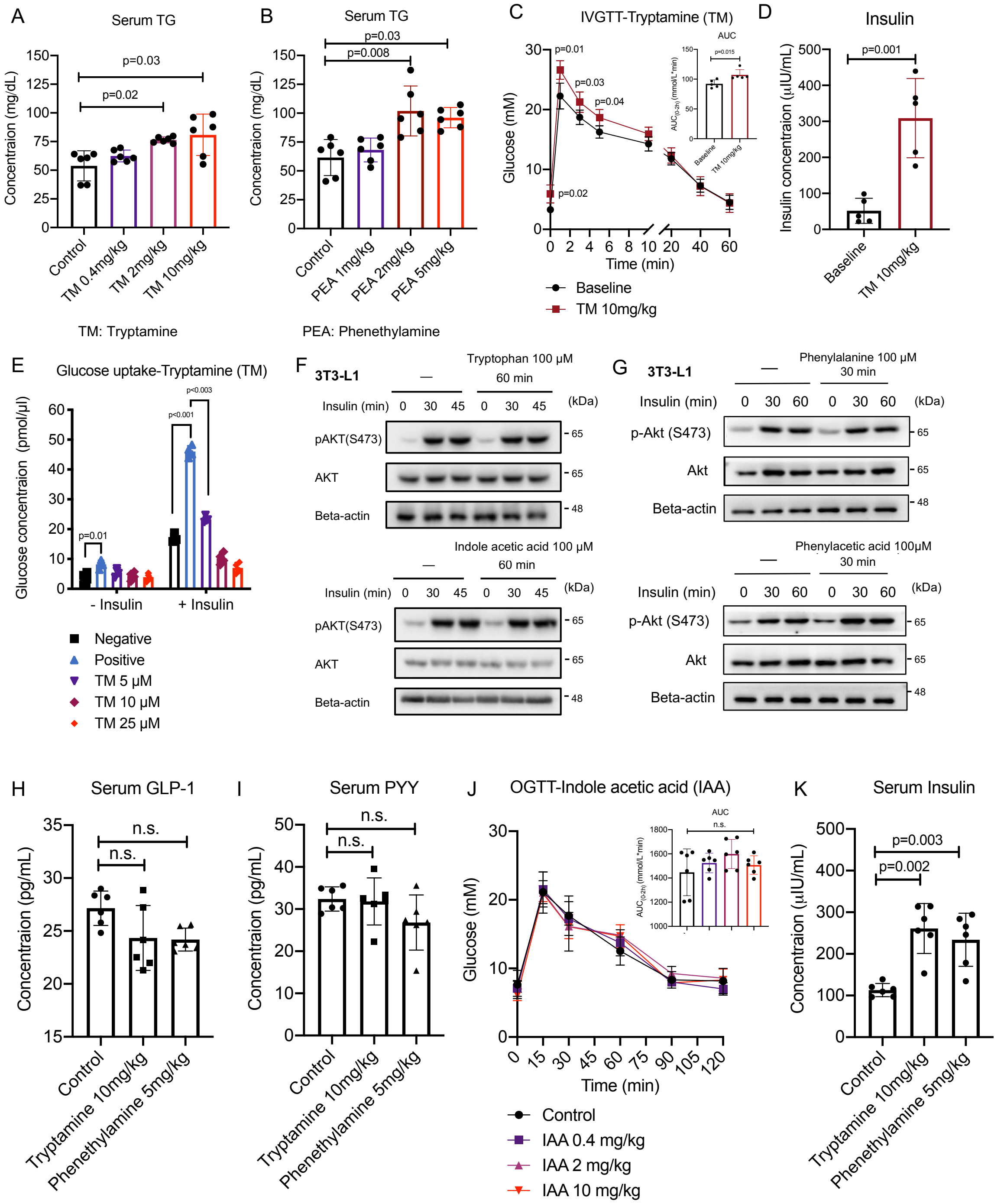

Figure.S4 Tryptamine and phenethylamine impair insulin sensitivity in mice, monkeys and *in vitro* models, Related to Figure.4

(A-B) Serum TG level in mice (n=6/group) after treatment with tryptamine or phenethylamine at indicated dosages or control (1% DMSO in saline) by i.p. **(C-D)** IVGTT index and serum insulin levels in monkeys after treatment with tryptamine (10mg/kg) or control (1% CMC-Na in water) (n=5/group). **(E)** Effect of tryptamine (5μM, 10μM and 25μM) on glucose uptake stimulated by insulin (20nM) in 3T3-L1 cells (n=3/group). **(F)** Western blot (and quantification) of tryptophan (100μM) and indole acetic acid (100μM) treatment (precursor and metabolite of tryptamine) on insulin signaling stimulated by insulin (20nM) in 3T3-L1 cells (n=3/group). **(G)** Western blot (and quantification) of phenylalanine (100μM) and phenylacetic acid (100μM) treatment (precursor and metabolite of phenethylamine) on insulin signaling stimulated by insulin (20nM) in 3T3-L1 cells (n=3/group). **(H-I)** Serum GLP-1 and PYY level in mice (n=6/group) after treatment with tryptamine or phenethylamine at indicated dosages or control (1% DMSO in saline) by i.p. (n=6/group). **(J)** OGTT index in mice after treatment with indole acetic acid at indicated dosages or control (1% DMSO in saline) by i.p. (n=6/group). **(K)** Serum insulin levels in mice after treatment with tryptamine (10mg/kg), phenethylamine (5mg/kg) or control (1% CMC-Na in water) (n=6/group). Data are presented as mean±S.D. *P* values were determined by two-tailed test of ordinary one-way ANOVA or Student's t-test.

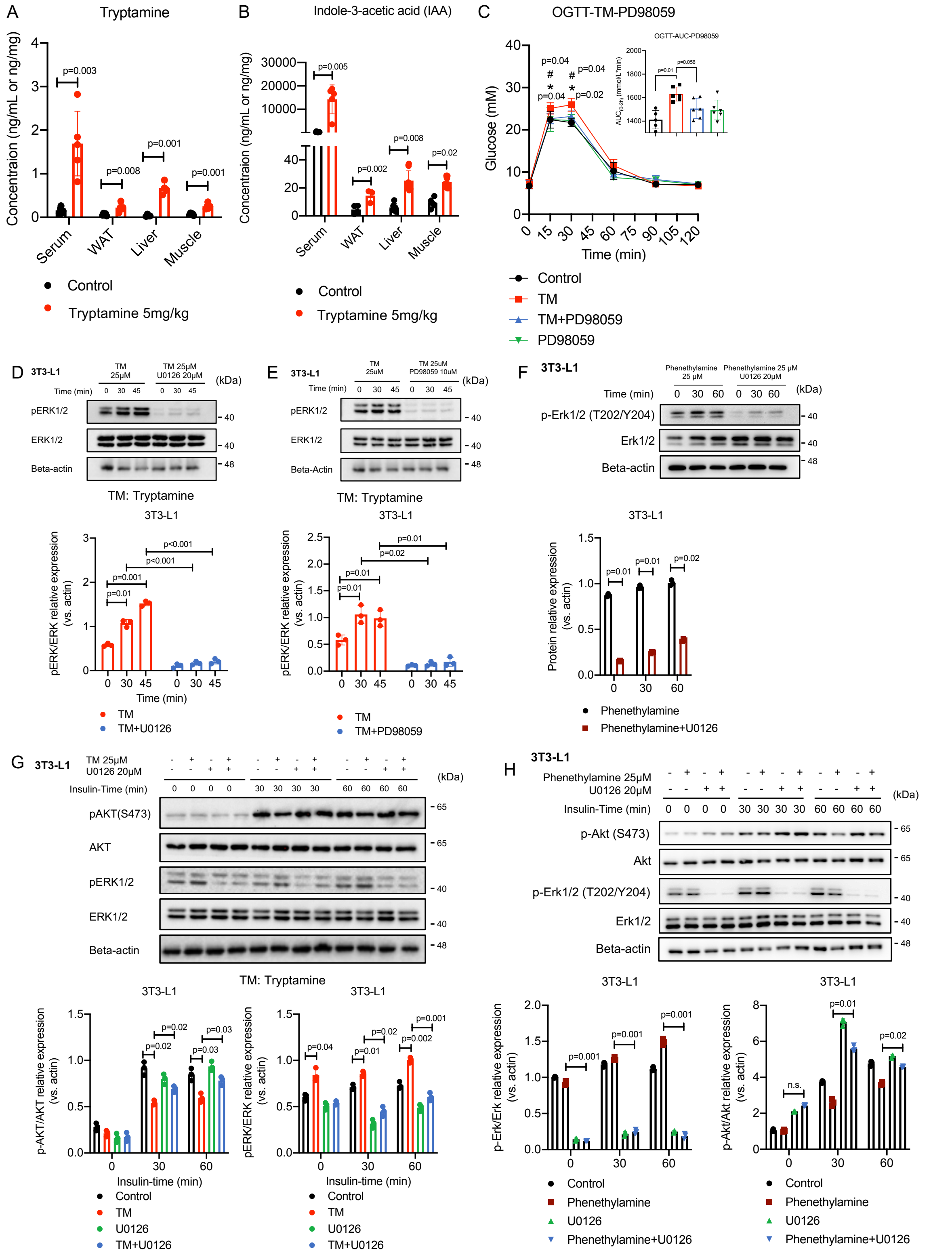

72

73 **Figure.S5 Tryptamine and phenethylamine impair insulin signaling via ERK activation**

(A-B) Tryptamine and indole acetic acid levels in serum, WAT, liver, and skeletal muscle after administration of tryptamine (5mg/kg) at indicated times (n=6). **(C)** OGTT index in mice after treatment of tryptamine (10mg/kg), ERK inhibitor PD98509 (10mg/kg) or control (1% DMSO in saline) (n=6/group). * Comparisons between control group and tryptamine group (10 mg/kg). $^{\#}$Comparisons between tryptamine group and tryptamine+ERK inhibitor (PD98059) group. **(D-F)** Western blot (and quantification) of the effect of tryptamine (25μM), phenethylamine (25μM) and ERK inhibitor U0126 on ERK activation in 3T3-L1 cells (n=3/group). **(G-H)** Western blot (and quantification) of the effects of tryptamine (25μM) and ERK inhibitor U0126 treatment (20μM) on ERK activation and insulin (20nM)-stimulated AKT activation in 3T3-L1 cells (n=3/group). Data are presented as mean±S.D. *P* values were determined by two-tailed test of ordinary one-way ANOVA or Student's t-test.

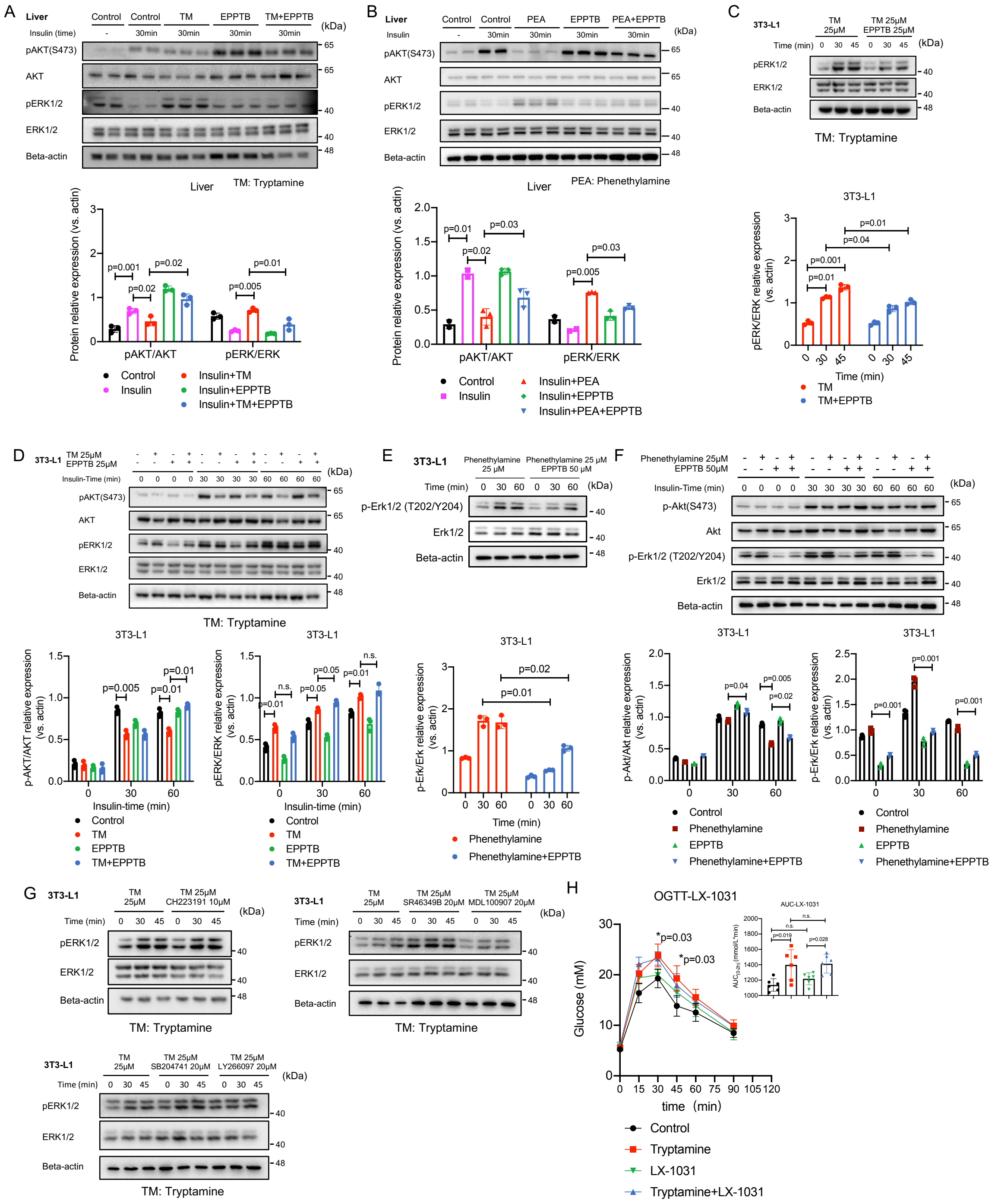

Figure.S6 Tryptamine and phenethylamine weaken insulin signaling via TAAR1-ERK signaling axis, Related to Figure.6

(**A-B**) Western blot (and quantification) of the effects of tryptamine (10mg/kg) and TAAR1 antagonist EPPTB (10mg/kg) treatment on ERK activation and insulin (1U/kg)-stimulated

AKT activation in liver lysates from mice (n=2 in control and insulin group, n=3 in other groups). **(C)** Western blot (and quantification) of the effects of tryptamine (25μM) and TAAR1 antagonist EPPTB treatment (20μM) on ERK activation in 3T3-L1 cells (n=3/group). **(D)** Western blot (and quantification) of the effects of tryptamine (25μM) and TAAR1 antagonist EPPTB treatment (20μM) on ERK activation and insulin (20nM)-stimulated AKT activation in 3T3-L1 cells (n=3/group). **(E)** Western blot (and quantification) of the effects of phenethylamine (25μM) and TAAR1 antagonist EPPTB treatment (20μM) on ERK activation in 3T3-L1 cells (n=3/group). **(F)** Western blot (and quantification) of the effects of phenethylamine (25μM) and TAAR1 antagonist EPPTB treatment (20μM) on ERK activation and insulin (20nM)-stimulated AKT activation in 3T3-L1 cells (n=3/group). **(G)** Western blot of tryptamine (25μM), AhR antagonist CH223191 (10μM), 5-HT2A receptor antagonist SR46349B (25μM) and MDL100907 (20μM) as well as 5-HT2B receptor antagonist SB204741 (20μM) and LY266097 (20μM) in 3T3-L1 cells (n=3/group). **(H)** OGTT index in mice after treatment of tryptamine (10mg/kg), TPH1 inhibitor LX-1031 (10mg/kg) or control (1% DMSO in saline) (n=6/group). * Comparisons between control group and tryptamine group (10 mg/kg). Data are presented as mean$\pm$S.D. *P* values were determined by two-tailed test of ordinary one-way ANOVA or Student's t-test.

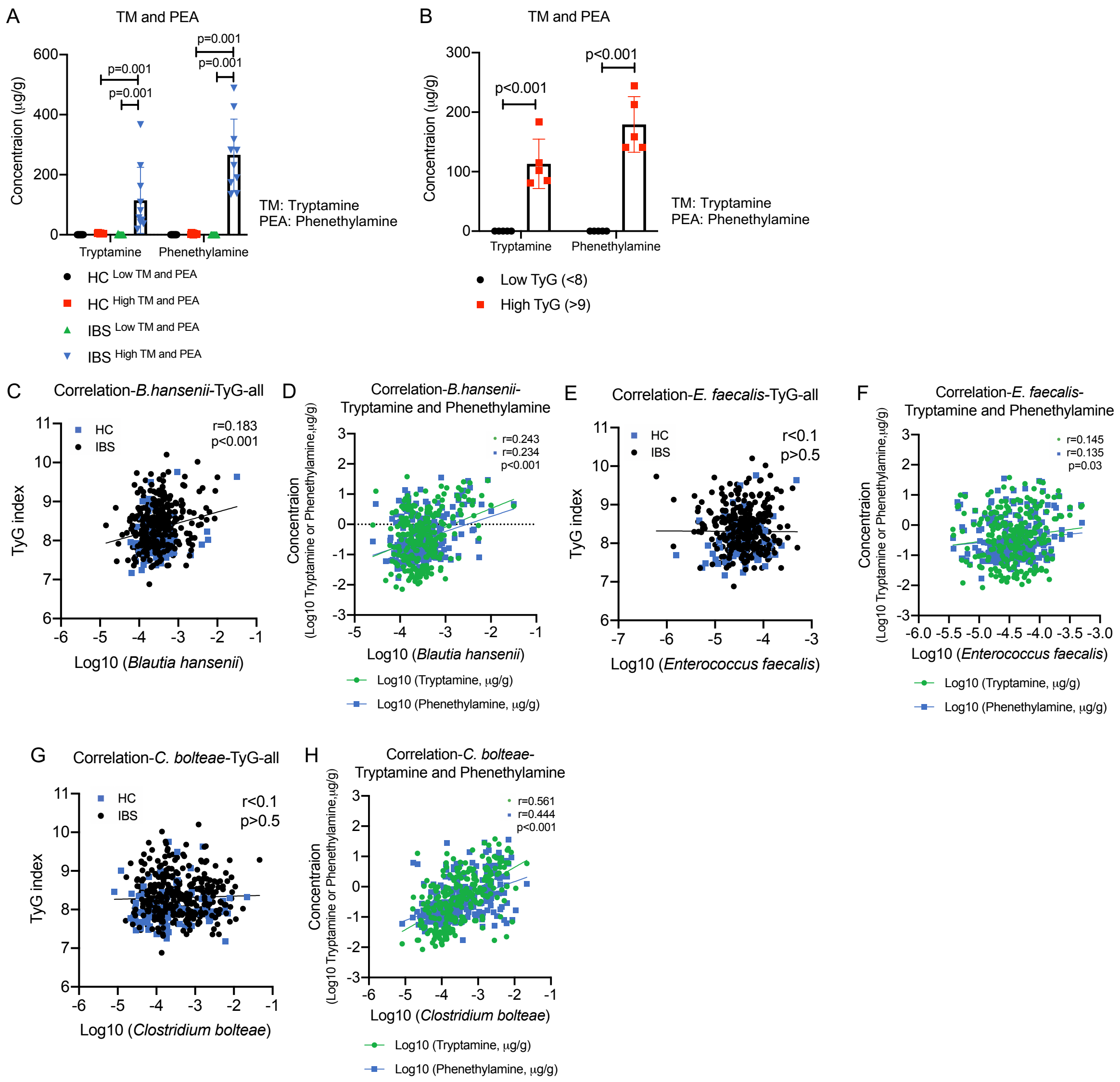

Figure.S7 TAAR1 inhibition alleviates insulin resistance induced by tryptamine and phenethylamine-producing bacteria

(A) Tryptamine and phenethylamine levels in fecal samples of HC and IBS subjects with low and high tryptamine and phenethylamine index (n=10/group). **(B)** Tryptamine and phenethylamine levels in fecal samples of HC and IBS subjects with low and high TyG index (n=5/group). **(C-D)** Spearman's correlation analysis between relative abundances of *Blautia hansenii* with TyG level and fecal tryptamine/phenethylamine level in human participants (n=412). **(E-F)** Spearman's correlation analysis between relative abundances of *Enterococcus*

faecalis with TyG level and fecal tryptamine/phenethylamine level in human participants (n=412). **(G-H)** Spearman's correlation analysis between relative abundances of *Clostridium boltae* with TyG level and fecal tryptamine/phenethylamine level in human participants (n=412). Data are presented as mean±S.D. *P* values were determined by one-tailed ordinary one-way ANOVA or student's t-test.
