# Supplementary material for: Gut microbiota-derived tryptamine and phenethylamine impair insulin sensitivity in metabolic syndrome and irritable bowel syndrome
Source: Nat Commun. 2023 Aug 17;14:4986. doi: 10.1038/s41467-023-40552-y (PMC10435514; doi:10.1038/s41467-023-40552-y)
Supplement: Supplementary file 3 — Description of Additional Supplementary Files [file 41467_2023_40552_MOESM3_ESM.docx]

Description of Additional Supplementary Files

File name: Supplementary Data 1

Description: BLASTP alignments with E-value < 1e-5 and identity > 30% to identify tryptamine producers in T2D subjects, Related to Figure.3

File name: Supplementary Data 2

Description: Correlation between fecal tryptamine with gut bacteria abundances in T2D subjects, Related to Figure.3

File name: Supplementary Data 3

Description: Phospho-peptides analysis of tryptamine effects on insulin-related pathways, Related to Figure.5

File name: Supplementary Data 4

Description: Reagent or Resource used in this study, Related to Methods

File name: Supplementary Data 5

Description: MRM transition and parameters used in targeted metabolomics, Related to Methods

File name: Supplementary Data 6

Description: Phospho-peptides identification of tryptamine treatment, Related to Figure.5

File name: Supplementary Data 7

Description: Phospho-proteins identification of tryptamine treatment, Related to Figure.5
