# Supplementary material for: Gut microbiota-derived tryptamine and phenethylamine impair insulin sensitivity in metabolic syndrome and irritable bowel syndrome
Source: Nat Commun. 2023 Aug 17;14:4986. doi: 10.1038/s41467-023-40552-y (PMC10435514; doi:10.1038/s41467-023-40552-y)
Supplement: Supplementary file 5 — Reporting summary [file 41467_2023_40552_MOESM5_ESM.pdf]

nature portfolio

Corresponding author(s): Zhaoxiang Bian, Liping Zhao, Wei Jia

Last updated by author(s): 2023, July, 11

Reporting Summary

Nature Portfolio wishes to improve the reproducibility of the work that we publish. This form provides structure for consistency and transparency in reporting. For further information on Nature Portfolio policies, see our Editorial Policies and the Editorial Policy Checklist.

Statistics

For all statistical analyses, confirm that the following items are present in the figure legend, table legend, main text, or Methods section.

n/a	Confirmed	
☐	☒	The exact sample size (n) for each experimental group/condition, given as a discrete number and unit of measurement
☐	☒	A statement on whether measurements were taken from distinct samples or whether the same sample was measured repeatedly
☐	☒	The statistical test(s) used AND whether they are one- or two-sided<br>*Only common tests should be described solely by name; describe more complex techniques in the Methods section.*
☐	☒	A description of all covariates tested
☐	☒	A description of any assumptions or corrections, such as tests of normality and adjustment for multiple comparisons
☐	☒	A full description of the statistical parameters including central tendency (e.g. means) or other basic estimates (e.g. regression coefficient) AND variation (e.g. standard deviation) or associated estimates of uncertainty (e.g. confidence intervals)
☐	☒	For null hypothesis testing, the test statistic (e.g. F, t, r) with confidence intervals, effect sizes, degrees of freedom and P value noted<br>*Give P values as exact values whenever suitable.*
☒	☐	For Bayesian analysis, information on the choice of priors and Markov chain Monte Carlo settings
☒	☐	For hierarchical and complex designs, identification of the appropriate level for tests and full reporting of outcomes
☐	☒	Estimates of effect sizes (e.g. Cohen's d, Pearson's r), indicating how they were calculated

Our web collection on statistics for biologists contains articles on many of the points above.

Software and code

Policy information about availability of computer code

Data collection	The metabolomics MS/MS spectra data were collected and analyzed using Agilent MassHunter Workstation Software.<br>Protein bands of western blots were quantified using Image J (version 1.51).<br>The proteomics MS/MS spectra were processed by Proteome Discoverer (Thermo Fisher Scientific) and searched using MASCOT engine 2.6.
Data analysis	Data analysis was performed using GraphPad Prism 8 and p-values less than 0.05 are considered statistically significant.<br>KEGG pathway annotation was performed using KOALA (KEGG Orthology And Links Annotation) to identify the significantly enriched pathways.

For manuscripts utilizing custom algorithms or software that are central to the research but not yet described in published literature, software must be made available to editors and reviewers. We strongly encourage code deposition in a community repository (e.g. GitHub). See the Nature Portfolio guidelines for submitting code & software for further information.

Data

Policy information about availability of data

All manuscripts must include a data availability statement. This statement should provide the following information, where applicable:

- Accession codes, unique identifiers, or web links for publicly available datasets
- A description of any restrictions on data availability
- For clinical datasets or third party data, please ensure that the statement adheres to our policy

All data supporting the findings in this study are available within the Article and Supplementary Information. Source data are provided as a Source Data file with this paper. Further information and requests for resources can be directed to and will be fulfilled by Zhao-Xiang Bian (bzxiang@hkbu.edu.hk). Fecal metagenomic sequencing data of the IBS study40 can be obtained from CNGB Nucleotide Sequence Archive (https://db.cngb.org/cnsa/) under accession number CNP0000334. Fecal metagenomic sequencing data of GUT2D study20 can be obtained via the European Nucleotide Archive under accession numbers PRJEB14155.

Research involving human participants, their data, or biological material

Policy information about studies with human participants or human data. See also policy information about sex, gender (identity/presentation), and sexual orientation and race, ethnicity and racism.

Reporting on sex and gender	We have added sex and gender considerations in this study including human and vertebrate animals' experiments. We included both male and female subjects in our clinical studies so that we can determine the changes of tryptamine, phenethylamine and gut microbiota in both genders. We apply the tryptamine and phenethylamine to male mice and male monkeys only to study the effects of tryptamine and phenethylamine on insulin sensitivity as proof of concept evidence. No gender-based analyses were performed in the animal study as we found the elevation of tryptamine and phenethylamine are increased in both male and female. Therefore, we consider the dysbiosis of gut microbiota affects both genders and tryptamine and phenethylamine levels are increased in both male and females.
Reporting on race, ethnicity, or other socially relevant groupings	No race, ethnicity or other socially relevant groups information were used or analyzed in this study.
Population characteristics	No obvious co-variate factors including age, sex, past diagnosis and treatment were found in this study
Recruitment	Participants were recruited according to the study protocol in each project. The recruitment was conducted via social media and advertisement. No obvious self-bias selection was found in this study.
Ethics oversight	The first cohort including healthy controls and IBS subjects was approved by the Research Ethics Committee of Hong Kong Baptist University (Ref ID:HASC/15-16/0300 and NCT02822677).<br>The second cohort including healthy controls and T2D subjects was approved by the Research Ethics Committee of Shanghai Jiao Tong University Affiliated Sixth People's Hospital.<br>The third GUT2D study was approved by the Ethics Committee at the School of Life Sciences and Biotechnology, Shanghai Jiao Tong University (Ref ID: 2014-016/ChiCTR-TRC-14004959).

Note that full information on the approval of the study protocol must also be provided in the manuscript.

Field-specific reporting

Please select the one below that is the best fit for your research. If you are not sure, read the appropriate sections before making your selection.

☒ Life sciences ☐ Behavioural & social sciences ☐ Ecological, evolutionary & environmental sciences

For a reference copy of the document with all sections, see nature.com/documents/nr-reporting-summary-flat.pdf

Life sciences study design

All studies must disclose on these points even when the disclosure is negative.

Sample size	For in vivo studies, we calculated the sample size, based on our extensive experiences with animal models and endpoints. Proper sample sizes (N=6) were chosen to generate reproducible results with desirable significance (<0.05) and power (>90%).<br>For in vitro experiments, sample sizes (at lease three independent experimental replicates) were chosen based on the standard practice of the research field (N=3) to generate reproducible results with desirable significance (<0.05) and power (>90%).<br>We have included sex and gender considerations in this study including human and vertebrate animals' experiments. Both male and female human subjects were involved for the measurement of gut microbes and microbial metabolites.
Data exclusions	No data was excluded from the manuscript.
Replication	For in vivo experiments, experimental results were replicated as indicated in figure legends e. For in vitro experiments, each experiment was independently repeated at least three times. Biological replicates were plotted and used for statistical analyses. We confirmed that all attempts at replication were successful in this study.
Randomization	Prior to treatment, mice were randomized and separated into different groups matched on body weight. For in vivo mice experiments, tissues

	from independently and randomly chosen mice were collected for analyses and none of the samples was excluded. For in vitro experiments, groups were allocated based on the different treatments so no randomization was required.
Blinding	Glucose tolerance test and insulin sensitivity testing experiments were performed blinded. For other in vivo and in vitro experiments. the investigator were not blinded during data collection or analysis. This approach is considered standard for experiments of the type performed in this study, as the genetic background of mice or treatment must be predetermined prior to analysis.

Reporting for specific materials, systems and methods

We require information from authors about some types of materials, experimental systems and methods used in many studies. Here, indicate whether each material, system or method listed is relevant to your study. If you are not sure if a list item applies to your research, read the appropriate section before selecting a response.

Materials & experimental systems

n/a	Involved in the study	
☐	☒	Antibodies
☐	☒	Eukaryotic cell lines
☒	☐	Palaeontology and archaeology
☐	☒	Animals and other organisms
☐	☒	Clinical data
☒	☐	Dual use research of concern
☒	☐	Plants

Methods

n/a	Involved in the study	
☒	☐	ChIP-seq
☒	☐	Flow cytometry
☒	☐	MRI-based neuroimaging

Antibodies

Antibodies used	Rabbit anti-phospho-Akt (Ser473) Cell Signaling Cat# 4058 1:1000<br>Rabbit anti-Akt (1:1000) Cell Signaling Cat# 9272 1:1000<br>Rabbit anti-phospho-IRS-1 (Ser307) Cell Signaling Cat# 2381 1:1000<br>Rabbit anti-IRS-1 (D23G12) Cell Signaling Cat# 3407 1:1000<br>Rabbit anti-IRS-2 Cell Signaling Cat# 4502 1:1000<br>Rabbit anti-phospho-IRS2 (Ser731) Abcam Cat# 3690 1:1000<br>anti-β-Actin Cell Signaling Cat# 4970 1:2500<br>Rabbit anti-phospho-p44/42 MAPK Cell Signaling Cat# 9101 1:1000<br>Rabbit anti-p44/42 MAPK Cell Signaling Cat# 4695 1:1000<br>Rabbit anti-Insulin Receptor β (4B8) Cell Signaling Cat# 3025 1:1000<br>Rabbit anti-Phospho-Insulin Receptor β (Tyr1345) Cell Signaling Cat# 3026 1:1000<br>Goat anti-rabbit antibody conjugated with HRP sc-2030, Santa Cruz 1:5000<br>Rabbit anti-mouse antibody conjugated with HRP sc-358914, Santa Cruz, 1:5000
Validation	These antibodies were validated by their manufactures for Western blotting in mice and the detailed information can be found on the manufactures' websites.

Eukaryotic cell lines

Policy information about cell lines and Sex and Gender in Research

Cell line source(s)	3T3-L1 cells were purchased from ATCC (https://www.atcc.org/products/cl-173)
Authentication	Cell lines were not authenticated.
Mycoplasma contamination	The cells were tested negative for mycoplasma contamination
Commonly misidentified lines<br>(See ICLAC register)	No commonly misidentified cell lines were used

Animals and other research organisms

Policy information about studies involving animals; ARRIVE guidelines recommended for reporting animal research, and Sex and Gender in Research

Laboratory animals	Wild-type male mice on C57BL/6J background were from the Laboratory Animal Services Centre of The Chinese University of Hong Kong. All animal experiments involves animals with starting age of 6 weeks. All animals were housed in the Animal House at Hong Kong Baptist University and maintained on a 12-hour (h) light/dark cycle with constant ambient temperature (22°C – 24°C) and humidity (~60%).

	For the first crab-eating macaques (Macaca fascicularis) study, 78 male monkeys age from 10-23 years old were employed in this study.<br><br>For the second crab-eating macaques (Macaca fascicularis) study, 5 male monkeys age from 11-21 years old were employed in this study.
Wild animals	No wild animals were used in this study.
Reporting on sex	We have included sex considerations in mice and monkeys experiments and decided to use male mice and male monkeys to study the effects of trytapmine and phenethyalmine on glucose tolerance and insulin sensitivity as male mice are less affected by sex hormones which are also involved in the glucose control.
Field-collected samples	No field-collected samples were used in this study.
Ethics oversight	The first crab-eating macaques (Macaca fascicularis) metabolomics study is performed with ethics approval (No. YMB1704) by research ethnics committee of Yunnan Yinmore Biotechnology company (Kunming, China).<br><br>The second crab-eating macaques (n=5, 11-21 years old) intervention study is performed with ethics approval (No. HZ2021047) by research ethnics committee of Huazhen Biosciences company (Guangzhou, China).

Note that full information on the approval of the study protocol must also be provided in the manuscript.

Clinical data

Policy information about clinical studies
All manuscripts should comply with the ICMJEguidelines for publication of clinical research and a completedCONSORT checklist must be included with all submissions.

Clinical trial registration	The IBS clinical trail is registered in ClinicalTrials.gov (NCT02822677)<br>The diabetes clinical trial is registered in the Chinese Clinical Trial Registry (ChiCTR-TRC-14004959).
Study protocol	The IBS clinical study protocol can be found at https://clinicaltrials.gov/study/NCT02822677<br>The diabetes study protocol can be found at https://www.chictr.org.cn/hvshowproject.aspx?id=7264
Data collection	The IBS clinical trail For the clinical trial of IBS<br><br>settings and places<br>Chinese Medicine Clinics and Research Lab of School of Chinese Medicine, Hong Kong Baptist University<br><br>periods of time for recruitment and data collection<br>July 4, 2016 to January 30, 2017<br>Data collection and study details can be found via following link DOI: 10.1172/JCI130976<br><br>For the clinical trial of T2D<br>settings and places<br>Shanghai First People's Hospital and Shanghai Jiao Tong University<br><br>periods of time for recruitment and data collection<br>2014-07-21 To 2015-07-20<br>Data collection and study details can be found via following link DOI: 10.1126/science.aao5774 in resources section
Outcomes	For the clinical trial of IBS<br><br>Primary Outcome Measures:<br>the Distribution of IBS Subtype and their Chinese medicine Pattern in Hong Kong Population.<br><br>Secondary Outcome Measures:<br>the Host-gut Microbiota Metabolic Interactions Associated with IBS Subtypes<br>the Relationship Between Chinese medicine Pattern and Their Microbiota<br>the relationship between Chinese medicine pattern and metabolic profiling<br><br>Measurement<br><br>1) diagnosis of IBS(ROME 4); 2) age requirement of 18 to 65 years; 3) Measurement of IBS Symptom Severity Scale (IBS-SSS) > 75 points (a range of 0-500 points of VAS on five questions) at baseline and during the 2-week run-in period; 4) Evaluation of colonoscopy or barium enema within 5 years; 4) Gut microbiota function , composition and metabolism measured by LC-MS-based metabolomics and metagenomics.<br><br>For the clinical trial of T2D<br><br>Primary Outcome Measures: HbA1c

Secondary Outcome Measures: OGTT Glucose, OGTT Insulin, HOMA-IR index, HOMA-beta Index, Glycated Serum Protein, Cholesterol, Triglyceride, Low Density Lipoprotein, High Density Lipoprotein, Weight, Body Mass Index, Waistline, Hipline, Body Fat Distribution, Blood Pressure, CEA , Drug Usage , Intestines secrete peptide, Gut microbiota function , compositioin and metabolism of gut microbiota

Measurement

Biochemical parameters were determined by biochemical analyser, UPLC-MS or ELISA at the Shanghai General Hospital, Shanghai Jiao Tong University School of Medicine. Food intake, body weight, drug use (other than acarbose), fasting and 2-h postprandial blood glucose (Accu-Chek® Performa, Roche, Mannheim, Germany) and adverse events were self-reported. Gut microbiota composition and function were anlyzed by metagenomics seqneuncing using Illumina HiSeq 3000
